# Components of Service Delivery Models of Care for the Detection, Care and Management of Visual Impairment for Children with Cerebral Visual Impairment: A Scoping Review Protocol

**DOI:** 10.22599/bioj.498

**Published:** 2026-02-10

**Authors:** Lauren R. Hepworth, Ffion Curtis, Michelle Maden, Cathy Williams, John Ravenscroft, Shelley Robinson, Brinton Helliwell, Charlotte Croft, Andrew Hill, Ruaraidh Hill, Catrin Tudur Smith, Fiona J. Rowe

**Affiliations:** 1 Institute of Population Health, University of Liverpool, UK; 2 Liverpool Evidence Synthesis Group (LENS)/Liverpool Reviews and Implementation Group (LRiG), University of Liverpool, UK; 3 Bristol Medical School and Bristol Eye Hospital, Bristol, UK; 4 The Scottish Sensory Centre, Moray House School of Education and Sport, University of Edinburgh, UK; 5 Specialist Teaching and Learning Sensory Service, Kent County Council, UK; 6 VISable, Liverpool, UK; 7 University of Liverpool Management School, University of Liverpool, UK

**Keywords:** Cerebral visual impairment, CVI, child, education, evidence synthesis, scoping review, health services

## Abstract

**Introduction:**

Cerebral visual impairment (CVI) is an umbrella term that describes a broad range of brain related visual problems and it is now the most common cause of visual impairment (VI) in children in the developed world. Early assessment, management and support are essential to minimise the risk of lifelong negative consequences. Currently there are no internationally agreed clinical guidelines for the investigation and diagnosis of CVI, with variation in existing models of care resulting in potential service health inequities for this population.

**Objective:**

The objective of this scoping review is to identify and describe the components of existing service delivery models of care in relation to the detection, care, and management of children with CVI.

**Methods:**

We will search multiple databases to include MEDLINE, CINAHL, Embase, APA PsycINFO, and The Cochrane Library for English language publications from OECD countries. Supplementary searches will also be conducted to locate grey literature. We will include any study that describes or assesses service delivery for children (0–17 years) with CVI in health and social care, and education settings.

**Stakeholder engagement:**

We consulted with a broad range of stakeholders including subject experts and patient and public representatives during the planning and will continue during the conduct, reporting and dissemination of this scoping review.

## Introduction

Cerebral visual impairment (CVI) is an umbrella term that describes a broad range of brain related visual problems (Pilling and Ravenscroft, 2022) resulting from abnormal brain development or damage to the brain (McConnell, Saunders and Little, 2021). Whilst CVI can be acquired at any point during childhood, conditions leading to CVI often occur perinatally and are frequently reported in children born prematurely (McConnell, Saunders and Little, 2021). The incidence of CVI is increasing predominantly both due to advances in medical care and increased knowledge and awareness of CVI. CVI is now the most common cause of visual impairment (VI) in children in the developed world (Chang *et al*., 2024). Despite this, intervention research relating to CVI is still in its infancy in terms availability and quality of data (Delay *et al*., 2023).

It is well documented that VI has a considerable impact on the lives of not only those with VI, but also on their families and care givers (McDowell, 2021). In addition to quality-of-life issues, VI in children impacts numerous developmental domains to include development of motor skills, socialisation, and learning with the potential for lifelong consequences (Chokron and Dutton, 2023; Lueck *et al*., 2023). Significant health inequalities exist for this population (Bountziouka, Cumberland and Rahi, 2017; Teoh *et al*., 2023) with substantial barriers evident in relation to the evaluation, diagnosis, treatment, and education of this growing population (Lueck *et al*., 2023).

CVI related vision problems have been reported in at least 3% of primary school children, including in 40% of children recognised as having a need for additional educational support, almost all of which were previously undiagnosed (Williams *et al*., 2021). Hospital eye care is often focused on acute signs and variances can occur after discharge where eye care is managed across a mix of secondary or community eye care services. Therefore, children with CVI may struggle to access appropriate assessment and management. Critically, because of the age and developmental immaturity of children, liaison is required among many professionals, family, and other services (e.g. education). CVI may be associated with reduced visual acuity and/or visual field awareness and variable day-to-day visual behaviour, plus other visual impairments. However, patients may not report visual symptoms. This may simply reflect a mild visual problem, young age, or more complex brain damage preventing the recognition of these visual symptoms (Fazzi *et al*., 2007; Hepworth *et al*., 2021). Hence, it is important to have appropriate early and accurate patient assessments and high-quality evidence-based eye care provision delivered in appropriate care settings.

The complexities and deficits associated with CVI warrant a comprehensive evaluation and treatment approach provided by a multidisciplinary team consisting of ophthalmologists, paediatricians, neurologists, orthoptists, optometrists, occupational therapists, and teachers of students with visual impairments (including qualified teachers of learners with visual impairment (QTVIs) and qualified teachers of learners with multi-sensory impairment (QTMSIs), among others (Delay *et al*., 2023). Integrated service provision requires diverse groups of professionals situated across a range of organisational settings to work collaboratively to provide care for complex cases.

Assessment and/or models of care can vary widely for this population. The literature on service-delivery models for detecting, caring for, and managing cerebral visual impairment (CVI) in children is growing but remains fragmented, with far more guidance and consensus statements than evaluated models of care. Recent consensus practice guidance, from the Royal College of Ophthalmologists, outline roles within multidisciplinary teams (ophthalmology, paediatrics, neurodisability, habilitation, education) and emphasize streamlined referral, functional assessment, and early supports, but stop short of prescribing a single, validated model of care (Pilling *et al*., 2023). At a global policy level, WHO’s Package of Eye Care Interventions and the Vision and Eye Screening Implementation Handbook provide primary-care and school-screening frameworks that can be adapted to include CVI, yet they offer broad service architecture rather than CVI-specific model testing (Keel *et al*., 2022). The 2024 American Academy of Paediatrics further codifies paediatric roles in CVI detection and coordinated management, reinforcing multidisciplinary pathways (Lehman, Yin and Chang, 2024). By contrast, the intervention evidence base remains sparse and heterogeneous underscoring the need for rigorous evaluations of integrated models linking detection to educational and rehabilitative supports.

In addition to the barriers at the individual level, there is a lack of clarity and consensus in terms of the assessment and models of care for children with CVI. Key issues identified include a lack of consensus with regards to the definition and diagnostic criteria of CVI, the need for valid and reliable assessment methods, narrow ocular/acuity-based classification of visual impairment across medical, education, and governing bodies, and the need for high quality evidence to inform interventions (Lueck *et al*., 2023). Previous reviews of CVI in children have tended to focus on specific areas such as assessment, diagnostic techniques, motor skills, functional vision (McConnell, Saunders and Little, 2021), or targeted interventions (Delay *et al*., 2023) as opposed to care pathways more broadly. Due to the deficits and complexities associated with CVI an integrated service provision (Croft *et al*., 2021) is required to address the assessment and treatment needs of this population. In their recent systematic review Delay *et al*., (2023) highlighted the infancy of research in this area. Aligning with the number three research priority for childhood onset, as set out by the UK clinical eye research strategy (Bourne *et al*., 2024), what is needed now is an overview of existing CVI service delivery around the integrated service provision for the detection and management of CVI to provide a better understanding of current practice (Bourne *et al*., 2024).

### Aims and objectives

The aim of this scoping review is to identify and report common elements of global CVI service delivery around the detection and management of visual impairment and integrated service provision (Croft *et al*., 2021), capturing care resources involved to scope potential care costs. This will enable us to systematically map research findings across a body of research evidence that is heterogeneous and/or complex in nature to inform future service delivery in the UK.

### Objectives

We plan to describe the content, configuration and setting of health and wider care resources involved global CVI service delivery. This will include describing the quantity, frequency, and duration of service consultations and appointments, and understanding and reporting the cost implications. We will also identify and map elements of integrated service provision of CVI service delivery models.

A preliminary search of MEDLINE, the Cochrane Database of Systematic Reviews and *JBI Evidence Synthesis* was conducted and no current or underway systematic reviews or scoping reviews on the topic were identified.

## Methods

The proposed scoping review will be conducted in accordance with the JBI methodology for scoping reviews (Peters *et al*., 2020). This protocol follows the Preferred Reporting Items for Systematic Reviews and Meta-Analyses (PRISMA) reporting guidelines for protocols (Shamseer *et al*., 2015) and scoping reviews (Tricco *et al*., 2018). Patient and public representatives have helped shape the review question and will contribute the conduct and reporting of this scoping review.

### Identifying relevant studies

#### Eligibility criteria

A broad range of written sources will be considered to include research studies, systematic reviews, service evaluations, audits, guidance, quality standards or practice recommendations. Study protocols, conference abstracts, editorials, letters will be excluded.

#### Population

Children aged 0–17 years with CVI, carers of children with CVI, or professionals involved in the delivery of services for the CVI. We define CVI as verifiable visual impairment caused by disturbance of the central visual pathways whatever their mode of presentation (Sakki *et al*., 2018).

### Concept

CVI often causes vision loss, or variable vision. CVI may impact mental health, prevent children from pursuing certain occupations and the effect on appearance may interfere with psychosocial development (Bennett *et al*., 2024; MacKenzie *et al*., 2016). However, patients may not report visual symptoms. This may simply reflect a mild visual problem, young age or more complex brain damage preventing the recognition of these visual symptoms (Fazzi *et al*., 2007; Hepworth *et al*., 2021). Also, early signs and symptoms of CVI may be attributed to learning disability, autism or developmental delay, further delaying a diagnosis of any kind (Lehman *et al*., 2024). Early assessment, management and support is required to maximise the visual capabilities of a child and promote engagement and interaction with their environment. Delayed visual diagnosis and management for children carries lifelong consequences (Rahi *et al*., 1999).

### Context

Currently health inequalities exist where, for example, service provision is *ad hoc* and non-standardised across geographical regions (Bountziouka, Cumberland and Rahi, 2017; Teoh *et al*., 2023).

Service delivery models (detection, care, and management of VI): how services are delivered, organised and how they assess and manage patients with CVI will be included. These will include (but are not limited to), screening and specialist tests, settings of delivery, staff involvement, timing of assessment and follow-up, referral pathways and clinical and cost outcomes.

Studies conducted in hospital, community, educational, and home settings will be included. We will include studies from countries with membership of the Organisation for Economic Co-operation and Development (OECD).

Any study that describes or evaluates CVI service delivery around the detection or management of visual impairment (e.g. systematic reviews, randomised controlled trials, surveys, process evaluations, economic evaluations).

### Information sources

We will search MEDLINE, CINAHL, Embase, APA PsycINFO, Cost-Effectiveness Analysis (CEA), International HTA Database, ERIC, British Education Index, Education Research Complete, and The Cochrane Library (CENTRAL and CDSR) from inception onwards restricted to English language publications only.

Supplementary searches will also be undertaken for a UK focused search of grey literature such as: Health Management Information Consortium (HMIC), websites of selected relevant organisations e.g. RNIB, The CVI Society, SeeAbility, CVI Scotland etc. We will also conduct targeted Google Scholar, LENS.org and Web of Science searches (Booth *et al*., 2013). A call for evidence was also made to the clinical and educational communities involved the care of children with CVI for reports, service evaluations, audits, guidance, quality standards, practice recommendations and notice of ongoing research.

### Searches

A comprehensive and wide-ranging search will explore services across the spectrum of hospital, community, social care, education and other related settings for CVI.

An initial exploratory search will be developed in an iterative manner in MEDLINE from keywords relevant to CVI identified by the review team (to include an information specialist), stakeholder group and published topic-relevant systematic reviews. Keywords will include child terms AND vision disorder terms. Subject headings (e.g. MeSH), free text, and advanced search techniques (e.g. truncation, proximity operators) will be used. The initial search for each topic will evolve during discussions with the wider research team and stakeholder group to ensure that the search terms are relevant.

A sample of relevant records will be used to conduct sensitivity analysis on search terms to check the sensitivity of the searches and amend as required. An iterative approach to searching will be undertaken as our understanding of the literature increases. Once the searches are tested and validated in MEDLINE, we will translate them across other sources. No date or study design limitations will be applied to the search strategies, but we will exclude animal studies.

In addition, we will contact topic experts to seek unpublished/ongoing studies, check reference lists of included studies and carry out forward citation searching.

### Selection of evidence

Search results will be downloaded into a bibliographic record management system (e.g. EndNote) and de-duplicated before uploading for screening and selection using a review management platform (e.g. Rayyan). Studies will be selected for inclusion through a 2-stage process i.e. title/abstract screening followed by full text screening using the predefined explicit criteria. Title/abstract screening will be completed independently by two reviewers to exclude records that do not meet the inclusion criteria and select records that may meet inclusion criteria for further review at the full text stage. Full texts of those selected will be retrieved and independently screened by two reviewers against the inclusion criteria. Any disagreements on eligibility decisions will be resolved through consensus and, if necessary, by discussion with a third reviewer.

The results of the search and the study inclusion process will be reported in full in the final scoping review and presented in a Preferred Reporting Items for Systematic Reviews and Meta-analyses extension for scoping review (PRISMA-ScR) (Tricco *et al*., 2018).

### Data charting

A standardised data extraction form was developed and piloted. Data to be extracted will be informed by discussions with our stakeholder group. Data relating to study characteristics (e.g. author, date, country, study design), sample (e.g. patient demographics, sample size), models of service delivery (e.g. types of services/test/intervention provided, format, delivery, intensity, frequency, duration, underlying framework, or theories, etc.), and equity will be extracted by one reviewer and independently checked for accuracy by a second reviewer.

The draft data extraction tool will be modified and revised as necessary during the process of extracting data from each included evidence source. Modifications will be detailed in the scoping review. Any disagreements that arise between the reviewers will be resolved through discussion, or with an additional reviewer/s. If appropriate, authors of papers will be contacted to request missing or additional data, where required.

### Data analysis and presentation

A narrative synthesis of the descriptions of service delivery will be reported. We will highlight common elements of what reportedly works and what doesn’t work in relation to service delivery models. Statistics on the effects of service delivery models will be extracted as reported, but, where data allows, standardised in evidence tables (noting any calculations). To compare the economic results of individual studies, costs will be converted to 2024 GBP by applying the gross domestic product deflator index and purchasing parities conversion rates using the CCEMG-EPPI cost converter tool (Shemilt *et al*., 2010), and a narrative approach will be used to present these findings. Evidence will be summarised in tables and synthesised narratively (Campbell *et al*., 2020).

#### Stakeholder engagement

We will continue to consult with a broad range of stakeholders including subject experts (including but not limited to eye care professionals, therapists, QVTIs) and patient and public representatives during the planning, conduct, reporting and dissemination of this scoping review. Group members will be sought through advertisements via relevant charities (for example different strokes, headway) and professional organisations (for example British and Irish Orthoptic Society (BIOS), UK Neuro-Ophthalmology Society (UKNOS), Vision Impairment Education Workforce (VIEW), and word of mouth. We will have regular meetings and also share documents to receive feedback on all review tasks to include the scope of the review, search terms, the type of evidence to include, data presentation, reporting, and dissemination planning.

#### Dissemination

All data in this project will be secondary, gathered through database searching or call for evidence. We will develop our dissemination strategy with stakeholder input, which will include sharing our findings via social media (e.g. X, Bluesky), peer reviewed publications, existing networks, and stakeholder engagement meetings and events.

### Summary

For CVI, early assessment, management, and support is essential to maximise the visual capabilities of a child and promote engagement and interaction with their environment. Delayed visual diagnosis and management for children carries lifelong consequences (Rahi *et al*., 1999). with these conditions. Currently there are gaps in high quality evidence relating to the assessment and measurement of the psychosocial impact of these conditions, and to the organisation of health care to detect and manage these conditions, as well as how to better integrate care across NHS and partner services such as social services, education and charity sectors. This scoping review will identify and present much needed information on models of service delivery across the patient pathway for individuals with CVI.
